# Febrile Seizures and Subsequent Autism Spectrum Disorder: A Nationwide Population-Based Cohort Study

**DOI:** 10.3390/children13030411

**Published:** 2026-03-17

**Authors:** Ya-Hsuan Tsai, Go-Shine Huang, Mei-Hua Hu

**Affiliations:** 1 School of Medicine, College of Medicine, Chang Gung University, Taoyuan 333, Taiwan; 2 Department of Medical Education, Chang Gung Memorial Hospital at LinKou, Taoyuan 333, Taiwan; 3 Department of Anesthesiology, Tri-Service General Hospital, National Defense Medical University, Taipei 114, Taiwan; 4 Department of Anesthesiology, School of Medicine, College of Medicine, National Defense Medical University, Taipei 114, Taiwan; 5 School of Chinese Medicine, College of Medicine, Chang Gung University, Taoyuan 333, Taiwan; 6 Division of Pediatric General Medicine, Department of Pediatric, Chang Gung Memorial Hospital at LinKou, College of Medicine, Chang Gung University, Taoyuan 333, Taiwan

**Keywords:** autism spectrum disorder, febrile seizure, attention-deficit/hyperactivity disorder, epilepsy

## Abstract

**Highlights:**

**What are the main findings?**

• Febrile seizures were not independently associated with ASD risk after adjustment in a nationwide cohort.
• The elevated autism risk was associated with coexisting neurodevelopmental comorbidities rather than febrile seizures alone.

**What are the implications of the main findings?**

• Febrile seizures may serve as an early clinical marker of underlying neurodevelopmental vulnerability rather than a direct cause of autism spectrum disorder.
• Children with febrile seizures and neurodevelopmental comorbidities may warrant enhanced long-term developmental surveillance.

**Abstract:**

**Objectives:** To access the effects of febrile seizures from coexisting neurodevelopmental conditions that are commonly associated with autism spectrum disorder. We examined whether febrile seizures are independently associated with ASD after considering neurodevelopmental comorbidities and seizure-related clinical characteristics. **Methods:** We conducted a nationwide population-based matched cohort study using Taiwan’s National Health Insurance Research Database. The study included 948 children with FS and 3804 age- and sex-matched controls without FS. Participants were followed longitudinally for incident ASD. Associations were evaluated using Cox proportional hazards models with additional analyses restricted to the FS cohort. Neurodevelopmental comorbidities assessed included attention-deficit/hyperactivity disorder (ADHD), epilepsy, and Tourette syndrome/tic disorder. **Results:** Among 4752 children followed for more than 10 years, 43 (0.9%) developed ASD. FS were not independently associated with ASD in adjusted Cox regression models. In contrast, ADHD, epilepsy, and Tourette syndrome/tic disorder were strongly and consistently associated with ASD across analytic models. **Conclusions:** Febrile seizures were not independently associated with autism spectrum disorder. Instead, ASD risk was largely explained by coexisting neurodevelopmental comorbidities, consistent with a shared neurodevelopmental susceptibility framework. These findings suggest that developmental surveillance should prioritize children with neurodevelopmental disorders rather than those with febrile seizures alone.

## 1. Introduction

Autism spectrum disorder (ASD) is a complex neurodevelopmental condition characterized by impairments in social communication and interaction [1,2,3]. Its rising global prevalence reflects increased recognition, evolving diagnostic criteria, and contributions from genetic, perinatal, and environmental factors [2,3,4,5,6]. Children with ASD frequently have neurological and psychiatric comorbidities, particularly epilepsy and attention-deficit/hyperactivity disorder (ADHD) [1,2,3,4], suggesting shared neurodevelopmental mechanisms such as excitatory–inhibitory imbalance and neuroinflammation [4,7].

Febrile seizures (FS) are the most common seizure events in early childhood, affecting approximately 2–5% of children between 6 months and 6 years of age worldwide [8,9,10,11]. FS are generally considered benign and self-limited. However, children with FS have been reported to have higher rates of later epilepsy, cognitive impairment, and psychiatric disorders, raising the possibility that FS may occur in the context of underlying neurodevelopmental vulnerability [10,12,13,14].

The association between FS and ASD remains uncertain. Population-based studies have reported inconsistent findings, likely due to differences in study design, diagnostic criteria, or seizure classification [15,16]. In addition, seizure management–related factors have rarely been examined. Benzodiazepines such as diazepam are commonly prescribed for prolonged or recurrent FS and may reflect seizure severity or clinical complexity; however, their relationship with subsequent neurodevelopmental outcomes remains unclear.

Using Taiwan’s National Health Insurance Research Database (NHIRD), which provides nationwide coverage and longitudinal follow-up [17,18], we conducted a matched cohort study to assess whether febrile seizures are independently associated with subsequent ASD after accounting for major neurodevelopmental comorbidities and seizure management–related factors.

We hypothesized that febrile seizures do not independently increase the risk of ASD and that previously observed associations may reflect shared neurodevelopmental comorbidities. Therefore, the aim of this study was to evaluate the independent association between FS and ASD and to quantify the contribution of neurodevelopmental comorbidities and seizure-related clinical characteristics to ASD risk.

## 2. Materials and Methods

### 2.1. Data Source

This population-based retrospective cohort study used data from Taiwan’s NHIRD, which covers more than 99% of residents and provides comprehensive longitudinal records of outpatient visits, hospital admissions, procedures, and prescriptions. The database contains de-identified demographic information, diagnostic codes based on the International Classification of Diseases, Ninth Revision, Clinical Modification (ICD-9-CM), and details of healthcare utilization. This study was approved by the Institutional Review Board of Chang Gung Memorial Hospital (IRB No. 103-0779B), and informed consent was waived because all data were anonymized.

### 2.2. Study Population and Cohort Definition

Data were obtained from a representative cohort of 1,000,000 individuals randomly sampled from the NHIRD, comprising insured beneficiaries registered in 2005 and followed longitudinally. The study population included children aged 6 months to 6 years who were newly diagnosed with FS (ICD-9-CM code 780.31) between 1 January 1996, and 31 December 2011. Children were excluded if they had a prior diagnosis of epilepsy (ICD-9-CM 345.x), cerebral palsy (343.x), or were diagnosed with meningitis (320.x, 047.x, 049.x) or encephalitis (062.x, 323.x) within one month before or after the FS diagnosis. The index date was defined as the date of the first FS diagnosis. For each child with FS, four control children without a history of FS were randomly selected and matched by age, sex, and index year. Controls were assigned the same index date as their matched FS case. Participants were categorized into the febrile seizure cohort and the non-FS (control) cohort.

### 2.3. Outcome Definition

The primary outcome was incident ASD. ASD cases were identified using ICD diagnostic codes recorded in the NHIRD claims data. ASD was defined as ≥1 claim with an ASD diagnosis in any healthcare setting, including inpatient admissions or outpatient physician visits, using ICD-9-CM code 299.x (including autistic disorder and other pervasive developmental disorders). Only ASD diagnoses occurring after the index date were included. Participants were followed from the index date until the earliest ASD diagnosis, withdrawal from the insurance program, or 31 December 2011.

### 2.4. Covariates

Baseline covariates included demographic characteristics and comorbid conditions potentially associated with ASD risk. Neurodevelopmental comorbidities included epilepsy (ICD-9-CM 345.x), ADHD (ICD-9-CM 314.x), and Tourette syndrome/tic disorder (ICD-9-CM 307.20–307.23, 333.3), were included as baseline adjustment covariates and were not considered as time-varying variables. Participants with epilepsy or ASD diagnosed before the index febrile seizure were excluded to preserve temporal directionality. Other clinical conditions included allergic rhinitis (ICD-9-CM 477.x) and atopic dermatitis (ICD-9-CM 691.8). Seizure-related clinical factors included exposure to antiseizure medications, particularly diazepam and phenobarbital, identified from prescription records.

### 2.5. Statistical Analysis

Baseline characteristics were compared between participants with and without ASD using the chi-square test. Time-to-event analyses were performed using Cox proportional hazards regression models to estimate hazard ratios (HRs) and 95% confidence intervals (CIs) for the association between FS and incident ASD. Univariate Cox regression analyses were conducted for each covariate, followed by multivariable Cox regression models including FS status, demographic variables, neurodevelopmental comorbidities, seizure-related clinical factors, and socioeconomic variables. A subgroup analysis restricted to children with FS was performed to evaluate factors associated with ASD within this cohort. Statistical significance was defined as a two-tailed *p* value < 0.05. All statistical analyses were performed using SPSS version 22.0 (IBM Corp., Armonk, NY, USA). Forest plots were generated using GraphPad Prism version 8.0 (GraphPad Software, San Diego, CA, USA).

## 3. Results

### 3.1. Study Population

**Figure 1:**
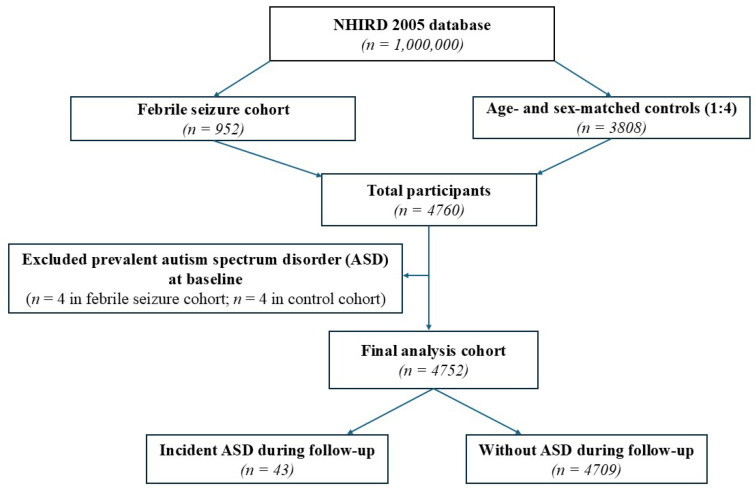
Flowchart of cohort selection from the National Health Insurance Research Database (NHIRD). From the NHIRD 2005 database, children with febrile seizures were identified and matched with controls without febrile seizures at a 1:4 ratio by age and sex. Individuals with a diagnosis of autism spectrum disorder prior to the index date were excluded. The final analysis cohort consisted of 4752 participants who were followed longitudinally for incident autism spectrum disorder.

After age- and sex-matching, 952 children with FS and 3808 matched controls without FS were included, yielding a total of 4760 participants. Following exclusion of children with a diagnosis of ASD prior to the FS diagnosis or index date, 4752 children comprised the final analytic cohort, including 948 children with FS and 3804 controls. The mean (standard deviation) age at cohort entry was 2.70 ± 1.22 years, and 58% of participants were male. During a mean follow-up of 12.34 years, 43 children (0.9%) were newly diagnosed with ASD (Figure 1).

### 3.2. Baseline Characteristics

Baseline demographic and clinical characteristics stratified by ASD status are shown in Table 1. Children who developed ASD had substantially higher prevalences of ADHD, Tourette syndrome/tic disorder, epilepsy, and diazepam exposure compared with children without ASD, whereas headache was less frequent among ASD cases. Similar patterns were observed within both the FS cohort and the non-FS control cohort.

### 3.3. Cox Proportional Hazards Analysis for ASD Risk

**Figure 2:**
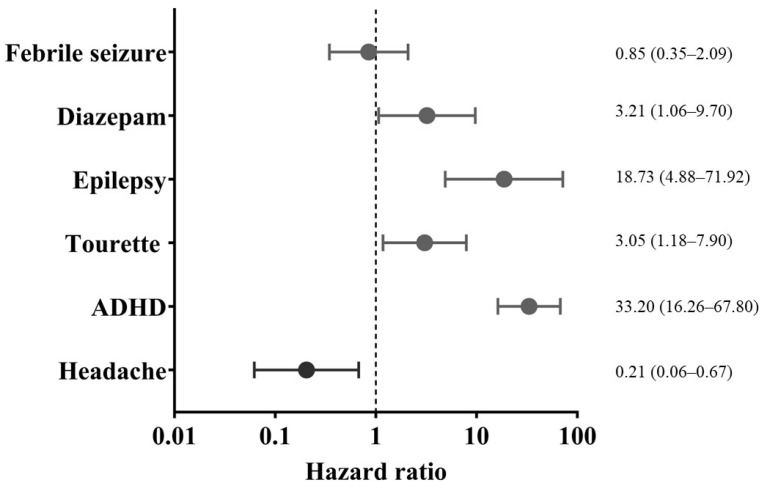
Forest plot of multivariable Cox regression results for new-onset autism spectrum disorder in the total study population. Points represent hazard ratios and horizontal bars indicate 95% confidence intervals. The vertical dashed line represents a hazard ratio of 1. Age, sex, and atopic dermatitis are not shown to improve visual clarity. All variables displayed were simultaneously included in the multivariable model.

Results of the Cox proportional hazards regression analyses are presented in Table 2 and summarized in Figure 2. In the multivariable model, febrile seizures were not independently associated with ASD (hazard ratio [HR] 0.85; 95% confidence interval [CI] 0.35–2.09). ADHD was associated with the highest risk of ASD (HR 33.20; 95% CI 16.26–67.80), followed by epilepsy (HR 18.73; 95% CI 4.88–71.92) and Tourette syndrome/tic disorder (HR 3.05; 95% CI 1.18–7.90). Diazepam exposure remained independently associated with ASD (HR 3.21; 95% CI 1.06–9.70). Headache was inversely associated with ASD risk (HR 0.21; 95% CI 0.06–0.67).

### 3.4. Analysis Restricted to the Febrile Seizure Cohort

In analyses restricted to children with febrile seizures in Table 3, ADHD remained a strong predictor of ASD (HR 38.98, 95% CI 9.98–152.31), epilepsy (HR 16.30, 95% CI 3.52–75.48) and Tourette syndrome / tic disorder (HR 6.25, 95% CI 1.25–31.34). Sex, age at index date, recurrent febrile seizures, headache, and atopic dermatitis were not significantly associated with ASD risk in this subgroup. Diazepam use showed an elevated hazard ratio (HR 2.80, 95% CI 0.89–8.80) but did not reach statistical significance. Overall, within the febrile seizure cohort, ASD risk was predominantly associated with coexisting neurodevelopmental comorbidities rather than seizure recurrence or diazepam use.

## 4. Discussion

In this nationwide population-based cohort study, neurodevelopmental comorbidities—particularly ADHD, epilepsy, and Tourette’s syndrome/tic disorder—were strongly associated with ASD after adjustment. These findings support a neurodevelopmental vulnerability framework, indicating that autism risk among children with seizures reflects shared underlying neurodevelopmental liability rather than a direct effect of febrile seizures. This interpretation is supported by genetic and neurobiological evidence demonstrating overlapping risk loci related to synaptic signaling and cortical network regulation [19,20,21], as well as neurophysiological features such as increased cortical excitability and impaired inhibitory control observed in ADHD and ASD [22,23]. Together, these observations align with the spectrum connectivity hypothesis, which conceptualizes ADHD and ASD as related manifestations of shared neural network dysregulation [24].

Within the FS cohort, epilepsy remained a significant independent predictor of ASD. This pattern is consistent with prior evidence showing that epilepsy and ASD share overlapping neurodevelopmental vulnerabilities [15,25,26]. Although some children with FS may later develop epilepsy, the increased ASD risk appears to reflect underlying neurobiological susceptibility rather than the effect of FS itself. In this context, FS may reflect as an early clinical indicator of neurodevelopmental vulnerability rather than a causal contributor to ASD.

In contrast, our study found no independent association between febrile seizures and ASD. Given the recognized clinical heterogeneity of febrile seizures, recent evidence suggests that simple febrile seizures lasting longer than 6 min may reflect greater clinical heterogeneity and increased neurological risk [27]. Although seizure duration was not available in our database, this perspective provides an interpretive framework for understanding our population-based results.

This differs from the Swedish study, which reported a stronger association [28], potentially due to methodological differences, including twin-based sampling and interview-based assessments versus the use of administrative claims data and ICD-coded diagnoses in our nationwide cohort. These observations suggest that variation across studies may reflect methodological and population heterogeneity rather than true biological inconsistency.

Furthermore, our study identified an inverse association between headache and ASD, which may reflect differences in symptom reporting, as children with ASD may be less likely to communicate headache symptoms during clinical encounters. Therefore, headache likely reflects reporting behavior rather than a true predictor of ASD risk.

The observed association between diazepam use and ASD should be interpreted cautiously and may reflect confounding by indication, since benzodiazepines are preferentially prescribed for more severe febrile seizures. Experimental and registry-based evidence suggests potential neurodevelopmental effects related to GABA signaling and antiseizure medication exposure [29,30,31,32]. Further research is needed to clarify the role of benzodiazepine exposure in neurodevelopment.

The overlap among epilepsy, ADHD, and ASD supports the possibility of shared neurodevelopmental mechanisms involving neuroinflammation and excitatory–inhibitory imbalance [33,34]. Seizure-related inflammatory activation may disrupt neuronal migration and synaptogenesis [35], while recurrent neuronal excitation during sensitive developmental periods may destabilize cortical networks, contributing to atypical sensory processing and social cognition [36,37,38]. Together, these processes provide a potential framework linking seizure disorders and attentional dysregulation with ASD.

This study has several limitations. First, because the NHIRD is an administrative claims database, detailed maternal and perinatal factors (e.g., parental age, mode of delivery, medication exposure during pregnancy), clinical information regarding seizure severity, immune-related biomarkers, and genetic anomalies were not available for analysis. Second, reliance on claims-based diagnostic coding may influence outcome validity. Finally, Formal causal mediation analysis was not performed because precise temporal ordering and adequate control of mediator–outcome confounding, cannot be reliably satisfied in administrative datasets.

## 5. Conclusions

Febrile seizures do not appear to independently increase the risk of ASD. Rather, ASD risk is associated with underlying neurodevelopmental comorbidities, particularly ADHD and epilepsy. These findings support a shared neurodevelopmental vulnerability framework rather than a direct causal role of febrile seizures in ASD development. Developmental monitoring efforts should prioritize children with early neurodevelopmental disorders to enable timely identification and intervention.

## Figures and Tables

**Table 1:** Baseline characteristics of the study population stratified by autism spectrum disorder status.

Variables	Without ASD (n = 4709)	With ASD (n = 43)
Overall cohort	4709 (99.1)	43 (0.9)
Male	2717 (57.7)	36 (83.7)
Headache	1008 (21.4)	3 (7.0)
Atopic dermatitis	1433 (30.4)	20 (46.5)
ADHD	306 (6.5)	32 (74.4)
Tourette syndrome/tic disorder	73 (1.6)	5 (11.6)
Epilepsy	34 (0.7)	3 (7.0)
Diazepam	216 (4.6)	6 (14.0)
Febrile seizure cohort (n = 948)	935 (98.6)	13 (1.4)
Male	538 (57.5)	10 (76.9)
Atopic dermatitis	320 (34.2)	5 (38.5)
ADHD	73 (7.8)	10 (76.9)
Tourette syndrome/tic disorder	19 (2.0)	2 (15.4)
Epilepsy	29 (3.1)	3 (23.1)
Diazepam	216 (23.1)	6 (46.2)
Participants without febrile seizures (n = 3804)	3774 (99.2)	30 (0.8)
Male	2179 (57.7)	26 (86.7)
Atopic dermatitis	1113 (29.5)	15 (50.0)
ADHD	233 (6.2)	22 (73.3)
Tourette syndrome/tic disorder	54 (1.4)	3 (10.0)
Epilepsy	5 (0.1)	0 (0.0)

Values are presented as number (percentage). ASD = autism spectrum disorder; ADHD = attention-deficit/hyperactivity disorder.

**Table 2:** Cox proportional hazards regression analysis for new-onset autism spectrum disorder.

Variable	Univariate HR (95% CI)	*p* Value	Multivariable HR (95% CI)	*p* Value
Age	1.12 (0.88–1.42)	0.358	1.17 (0.91–1.49)	0.217
Male sex	3.80 (1.69–8.54)	0.001	1.97 (0.84–4.62)	0.12
Febrile seizure	1.75 (0.91–3.35)	0.093	0.85 (0.35–2.09)	0.724
Headache	0.27 (0.08–0.86)	0.027	0.21 (0.06–0.67)	0.009
Atopic dermatitis	2.00 (1.10–3.63)	0.024	1.75 (0.96–3.20)	0.069
Diazepam	3.29 (1.39–7.80)	0.007	3.21 (1.06–9.70)	0.038
ADHD	38.79 (19.55–76.56)	<0.001	33.20 (16.26–67.80)	<0.001
Tourette syndrome/tic disorder	7.90 (3.11–20.06)	<0.001	3.05 (1.18–7.90)	0.022
Epilepsy	9.80 (3.03–31.69)	<0.001	18.73 (4.88–71.92)	<0.001

Abbreviations: HR, hazard ratio; CI, confidence interval; ADHD, attention-deficit/hyperactivity disorder. All variables listed were entered into the multivariable Cox model.

**Table 3:** Univariate Cox proportional hazards regression analysis for new-onset autism spectrum disorder in the febrile seizure cohort.

Variable	HR (95% CI)	*p* Value
Male sex	1.58 (0.39–6.34)	0.522
Age	1.26 (0.82–1.92)	0.287
Recurrent febrile seizure	1.06 (0.32–3.51)	0.952
Diazepam use	2.80 (0.89–8.80)	0.079
Headache	0.17 (0.02–1.47)	0.107
Atopic dermatitis	1.71 (0.51–5.76)	0.384
ADHD	38.98 (9.98–152.31)	<0.001
Tourette syndrome/tic disorder	6.25 (1.25–31.34)	0.026
Epilepsy	16.30 (3.52–75.48)	<0.001

Abbreviations: HR, hazard ratio; CI, confidence interval; ASD, autism spectrum disorder; ADHD, attention-deficit/hyperactivity disorder; FS, febrile seizure. Each variable was analyzed separately using univariate Cox proportional hazards regression.

## Data Availability

The data presented in this study are available on request from the corresponding author. The data are not publicly available due to ethical restrictions.
